# Learning Last Hours of Life Care Through Patient Simulation Scenario: Experiences of Medical and Nursing Undergraduate Students

**DOI:** 10.1007/s40670-025-02457-x

**Published:** 2025-07-16

**Authors:** Sandra Rubio Bernabé, Leire Sevillano Garayoa, Amaia Urrizola Martínez, Ana Carvajal Valcárcel, Leire Arbea Moreno, Carlos Centeno Cortés, María Arantzamendi

**Affiliations:** 1 Hospital San Juan de Dios, Pamplona, Spain; 2 Nursing Care for Adult Patients Department, Faculty of Nursing, University of Navarra, Pamplona, Spain; 3 Innovation for a Person-Centred Care Research Group, Faculty of Nursing, University of Navarra, Pamplona, Spain; 4 Medical Education Department, University of Navarra, Pamplona, Spain; 5 Faculty of Nursing, University of Navarra, Pamplona, Spain; 6 University of Navarra Institute for Culture and Society, ATLANTES, Pamplona, Spain; 7 Navarra Institute for Health Research (IdiSNa), Pamplona, Spain; 8 Palliative Care Department, Clinic University of Navarra, Pamplona, Spain

**Keywords:** Last hours of life care, Simulated scenario, Palliative care, Medical/nursing education

## Abstract

**Background:**

Last hours of life (LHoL) care is crucial but often uncovered in training programs. Practical experience is essential for developing skills, and clinical simulations with standardized patients offer valuable learning opportunities. Despite their potential, research on LHoL simulations is limited. To address this gap, a LHoL simulated scenario (SS) was developed.

**Objective:**

To analyze medical and nursing students’ experiences after conducting a LHoL SS and its perceived impact on their professional development.

**Methods:**

A qualitative study was conducted with students enrolled in a Palliative Care course during 2021–2022. A total of 187 sixth-year medical and 129 fourth-year nursing students were invited; 180 participated (93 medical, 87 nursing). Data were collected using open-ended questions and analyzed through thematic analysis with triangulation by five researchers, comparing and contrasting the experiences of medical and nursing students.

**Results:**

Students’ experiences were categorized into three main themes: (a) an overall positive experience which elicited emotions, (b) a realistic experience in a safe environment, and (c) a useful “learning” experience for future professional practice. Related to learning outcomes, students highlighted the following: (a) understanding the importance of the contribution of professionals at the end of life, (b) emphasizing the humanistic perspective, (c) providing clinical guidance, and (d) exercising introspective self-knowledge.

**Conclusion:**

Completing a LHoL SS was defined as a positive experience by the students, providing a secure and reflective environment that offered a technical guide for addressing difficult situations while encouraging reflective engagement. This experience enhanced their awareness of human aspects above and beyond professional roles and offered practical guidance aligned with the goals of palliative care education and broader curricular standards.

**Supplementary Information:**

The online version contains supplementary material available at 10.1007/s40670-025-02457-x.

## Background

Providing end of life care is an inevitable aspect of patient care for all healthcare professionals, regardless of their specialty. Integrating Palliative Care (PC) into the undergraduate curriculum can help students understand the importance of end-of-life care and how to deliver it. However, the European Association of Palliative Care (EAPC) has noted that students receive limited theoretical education on end-of-life care [1–3], and opportunities for practical experience are scarce. Consequently, many healthcare professionals encounter the challenge of caring for dying patients and their families for the first time late in their clinical practice.

Experiential learning goes beyond traditional lectures by engaging students in active, hands-on experiences followed by structured reflection. This process fosters deeper understanding, allowing learners to reflect critically not only on their actions but also on their consequences. Widely supported by educational frameworks [4, 5], this approach is especially relevant in healthcare education, where students must not only acquire clinical knowledge but also develop essential professional skills.

Simulated scenarios (SS) are a key form of experiential learning, replicating clinical situations in a safe, controlled setting [6–8]. The inclusion of standardized patients enhances realism [9, 10], which not only facilitates the application of theoretical knowledge but also helps develop and refine professional skills that are challenging to teach through traditional lectures. As a result, SS have become essential in the training of health care students beyond technical competence, simulation-based experiences help shape the learner’s identity as a healthcare provider, deepening their understanding of what it means to be a doctor—not just what a doctor does [11].

Caring for patients in their last hours of life (LHoL) presents significant challenges, including managing symptoms, addressing the emotions of patients and their families, and handling one’s own emotions. Unfortunately, there is a lack of literature regarding the use of SS to train medical and nursing students in managing LHoL care.

To address this gap, a SS of LHoL involving standardized patients was developed and incorporated into the PC curriculum for medical and nursing students at the University of Navarra. The aim of this study was to explore the experience of undergraduate medical and nursing students following their participation in this scenario.

## Material and Methods

### Study Design

A qualitative generic study was conducted to explore the students’ experiences with the LHoL SS, taking into account their impressions and its possible contribution to the students’ learning. A qualitative approach was used to explore the experiences and perspectives of participants expressed in their own words [12].

### Context and Participants

The study was conducted with undergraduate medical and nursing students following the compulsory subject of PC at the University of Navarra in the academic year 2021–2022. The 3 European Credit Transfer and Accumulation System (ECTS) course takes place in the last year of each degree (the 6th year in the medical school and the 4th year in the nursing school). In the medical degree, the teaching is based on theoretical lessons, seminars, and SS. In the nursing degree, the training is based mainly on theoretical lessons. The SS of LHoL was introduced for the first time during the 2021–2022 academic year and was mandatory for all students attending the PC course.

### Recruitment

An invitation email was sent to all students after participating in the SS. It explained the study, stating that participation was anonymous and voluntary, and involved answering two open questions in a one-time Google form. No further follow-up or reminder to participate in the study was sent.

### The Last Hours of Life Simulated Scenario

The scenario consisted of a simulated LHoL case for students to learn how to deal with such situations.

The SS was performed by a standardized patient and family member and a real nurse. The case consisted of a patient with stage IV lung cancer experiencing liver failure after hyper progression. He is married and has two children. He is currently receiving PC at home delivered by a multidisciplinary team (a PC physician and a nurse). The patient’s wife is his sole companion, playing a key role by providing students with updates on the current status of the patient while also expressing her own suffering and need for care and compassion from the healthcare providers.

The scenario had a duration of 1 h and had two parts. In the first, the medical team (the students) performed a “home visit” to evaluate the patient’s condition as he displays signs of terminal delirium. The students were expected to diagnose and treat the delirium and explain the situation to the wife. In the second part, the students make a follow-up visit during which the patient dies. The students had to communicate the patient’s death to the wife and comfort her. The participants were divided into groups of 10 to 15 students to carry out the scenario: two students participated in each part of the scenario as the medical team while the rest watched the scenario in real time from another room.

The Medicine and Nursing scenarios shared the same structure but differed slightly in context. In Medicine, a real nurse played the nurse’s role in person, while in Nursing, actors portrayed the patient and family, and the physician was contacted by phone rather than being physically present (see Supplementary materials 1 and 2).

The main objective of the scenario was to learn how to accompany patients and their close ones in the last moments of life. This scenario was included in the curriculum of the PC subject in the degree of Medicine and Nursing.

### Data Collection

Data were collected from October 2021 to January 2022. After participating in the SS, all students received an email with a link to an informed consent and an online questionnaire. Those who consented to participate answered online to the following two open questions: (a) *Tell us briefly how was your experience in the scenario “Attending the last hours of life.”* (b) *In your* *opinion, what have you taken from this SS?* The form had no word limit. No identifiable details were collected.

All data were stored in accordance with the university’s standard data storage protocol, which complies with current Spanish data protection legislation (Organic Law 15/1999).

### Analysis and Rigor

The thematic analysis of open answers was conducted inductively. Five researchers (SR, AU, LS, MA, AC) read the students’ answers and independently coded them. Some researchers were involved in developing the scenario, while others were experts in qualitative research. Periodic meetings were held to share and discuss the coding, which was inductively grouped into subcategories, categories, and themes.

The researchers first analyzed data from the medical students, then proceeded to code the nursing data. When there were discrepancies, the original data were reviewed and a discussion was held to refine the categories and subcategories so as to remain faithful to what the students conveyed.

**Fig. 1:**
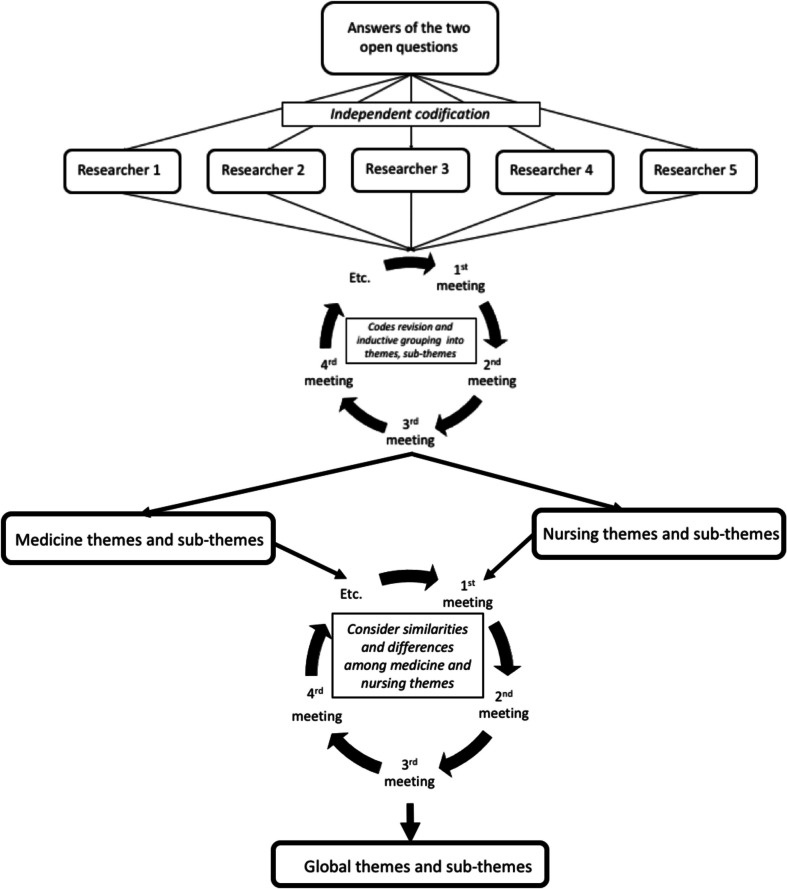
Inductive thematic analysis process conducted

The whole coding scheme was reviewed to consider similarities and differences among medical and nursing students (Fig. 1). Given the similarity of the findings, the results from both groups are presented together in the “Results” section, emphasizing the nuances or specificities. The quotes were used as an example of the categories. Reliability was obtained through researcher triangulation [12].

### Ethics Committee

The study was approved by the University of Navarra Clinical Research Ethics Committee (2012.193). All students provided informed consent before answering the questions.

## Results

A total of 316 students (187 from medicine and 129 from nursing) were invited to participate. Of these, 180 responses were completed, including 93 medical (50%) and 87 nursing (67%) students. Three responses were excluded as they were incomplete or referred to a different scenario.

The students’ answers concerning how their experience had been (students’ experiences) were grouped into three main themes: (a) an overall positive experience which elicited emotions and was an opportunity to learn emotional management; (b) a realistic experience in a safe environment; and (c) a useful “learning” experience for future professional practice; each theme includes sub-themes (Table 1).

**Table 1:** Themes and sub-themes of students’ experiences and attributed contributions of the last hours of life simulated scenario

Students’ experience (M = 93, N = 87)	Learning attributed to the scenario (M = 93, N = 87)
Overall positive experience which elicits emotions and is an opportunity to learn emotional management (M = 66, N = 40)	Understanding the importance of the contribution of professionals in the last hours of life (M = 44, N = 36)
- Positive experience of a difficult simulated scenario (M&N) - Experience that elicits uncomfortable emotions (M&N)	- Being at the patient’s and family side at the end of life is a substantial professional contribution (N) - Being human and not only dispensers of medical techniques (M) - Communicating bad news to the family empower them (M&N)
Realistic and enriching experience in a safe environment (M = 45, N = 42)	Emphasizing humanistic perspective: understanding (of) the emotions and needs (M = 18, N = 15)
- Safe environment that promotes the learning experience (M&N) - Realistic scenario that has an added value (M&N)	- Becoming aware of different types of needs in real life situations (M&N) - Acquiring essential knowledge on emotional management that cannot be found in the textbooks (M)
Useful learning experience for the future professional practice (M = 64, N = 57)	Providing guidance on how to act in delicate situations (M = 46, N = 60)
- Team building with nursing (M) - Enhances the importance of taking the family into consideration (M&N)	
	Exercising introspective self-knowledge (M = 15, N = 10)
	- Reflecting about their emotions when facing delicate situations and how to manage them (M&N) - Recognizing their own strengths and weakness (M&N) - Rediscovering palliative care (M&N)

In parentheses in the themes, the number of quotes from medical (M) and nursing (N) students that were part of it. In parentheses in the sub-themes, if the ideas were coded in medical (M), nursing (N), or both (M&N) students

Regarding what participants had taken from the scenario (learning attributed to the scenario), the students considered the main contribution to be the following: (a) understanding the importance of the professional contribution at the end of life, (b) emphasizing the humanistic perspective: understanding emotions and needs, (c) providing guidance on how to act in delicate situations and (d) exercising introspective self-knowledge.

## Students’ Experience

### An Overall Positive Experience Which Elicits Emotions and Is an Opportunity to Learn Emotional Management

Many students referred to the scenario as one that represented a delicate situation but emphasized that the *experience was positive*, as will be shown through the findings, *and was an opportunity to learn emotional management*. Despite the difficulty and stress, it was considered valuable for their development, with medical students more frequently highlighting the positive aspects than nursing students.

*“[…] Overall, I value the experience positively since we could see what it contributes and what it doesn't in these types of situations.” (M42)*

*“A very interesting experience in which we have to face a very complex situation that we have never faced before. I have learned a lot and I have prepared myself for the future.” (N52)*

Several medical and nursing students specified that the *experience elicited emotions*. Some were prompted to alleviate the actors’ perceived suffering, while others reflected on their own emotions, including *panic, stress, and being deeply moved*. Overall, the discourse remained mainly positive.

*“[...]At many times my legs trembled and tears came to my eyes. It was not easy for me, at times it reminded me of personal situations I had experienced.” (M57)*

*“It seemed to me a rather hard and human experience that as nurses we must go little by little knowing how to handle in order to help and accompany the patient and the family as much as possible.” (N35)*

### A Realistic and Enriching Experience in a Safe Environment

Some of the students, both in medicine and nursing, reported that this SS was perceived as an enriching, realistic experience in a *safe environment that facilitates learning.*

*“I found it to be a very enriching experience for us to be able to experience these situations from our practices and to be able to have tools for action when we have to experience it as health professionals.” (N29)*

*“It has been a complete and interesting experience that allows us to address a delicate scenario in a safe environment.” (M3)*

Students highlighted the *realism* of the activity as a valuable and innovative approach, distinct from theoretical learning about death. They felt immersed in the patient’s dying process, including caring for the family and managing both the family’s and their own emotions.

*“It seemed like a well-planned and worked out scenario, realistic and coherent. I think that it is the first time that we have carried out a scenario like this, with a patient wearing makeup […], it is a little shocking at first. However, this is a positive aspect of the simulated scenario because I think it achieves the objective of putting us in a real situation to try to handle it realistically” (M64)*

*“I found it to be a very interesting and necessary intervention, […]. The actors have helped make it seem like a real-life situation and I think it is a very good way to be able to “practice” what to do in a situation like this” (N47)*

### A Useful Learning Experience for Future Professional Practice

Most medical and nursing students indicated that the patient’s death SS was valuable for their future practice, as it reflects real professional experiences and enhances preparedness.

*“It seemed to me to be a necessary scenario. Undoubtedly it is a situation that we are going to encounter in practice and having this first contact in simulation brings you closer to how you could act in such a moment.” (N39)*

*“It has seemed to me a very useful scenario […]. It gives you the main idea of how to do things so if we have to deal with the situation we would not to be lost.” (M33)*

A few medical students noted it was their first opportunity to engage with such a situation in the course of their degree.

*“Very useful and interesting. It is great to learn how to act in such situations that we normally do not practice” (M87)*

Some medical students emphasized the importance of *team building* with nurses and appreciated the support network formed during the scenario. Nurses barely mentioned it.

*“[…] I think we have to learn to take nursing into account a little more, which sometimes we do not do and in this scenario its role has been essential.” (M17)*

Students from both professions emphasized the *importance of taking the family into account*. While nursing students viewed family care as an inherent part of holistic patient care, medical students saw it as a new perspective that highlighted the family member’s needs.

*“[…] Many of us have seen a patient in their last hours of life, but we have never had to face the difficulty of managing the emotions of family members, as well as ours. […]” (M10)*

*“[…] as nurses we must learn how to handle it [this kind of situation] in order to be able to help and accompany the patient and the family as much as possible.” (N35)*

## Learning Attributed to the Scenario

### Understanding the Importance of the Contribution of Professionals in the Last Hours of Life

Students recognized that *being by the patient’s and family side* in the LHoL is a significant professional contribution. Nursing students more explicitly noted that the scenario enhanced the humanistic aspect of their profession, valuing it more than in routine practice.

*“It has allowed me to enhance the human sensitivity and empathy, […]. I think it is due to the fact that in this case we stop practicing medicine for curative purposes and instead accompany and relieve the patient and the family with a holistic vision.”* (N74)

Medical students indicated that the scenario made them realize that medicine is not always about curing, but about always accompanying patients*.*

*“Mainly, this simulated scenario has given me perspective. Throughout our degree we are educated almost exclusively to “cure” diseases, cure patients. This means that when the patient dies, we think that our work is finished. Nothing is further from the truth; the doctor must be by the patient and their relatives in these last hours that are of vital importance for a complete and quality care.” (M24)*

*“After all, more than as ‘doctors’, we have to act as people.” (M11)*

Some students noted that the scenario highlighted the importance of *communicating with the family* and sharing information, even when *delivering bad news*. Providing such information empowers the family and supports them in their role as caregivers.

*“Knowing that the information we give to the family can bring them relief even if the news is bad and the family is suffering. Knowledgerelieves. There is no need to be afraid to tell the relative that her husband is dying.” (M93).*

*“Being aware that the family has a fundamental role in this situation and we must take care, support and inform about all the interventions that are going to be carried out, as well as the disease process and even the possibility of dying in the next few hours, […]” (N75).*

### Emphasizing the Humanistic Perspective: Understanding Emotions and Needs

Both student groups believed that the SS heightened their *awareness of the physical and emotional needs* of the individual in their care and emphasized the importance of addressing and managing these needs.

*“It has taught me […] how to manage and do things and seeing how patients feel.” (N5)*

*“It has allowed me to put myself in the doctor's shoes in that situation, and at the same way to see the family’s perspective. Identify what they need.” (M4)*

Some medical students pointed out that this is not taught in the textbooks, but it is *essential in real life*.

*“We know how to work with a simulated patient at the level of diagnosis and treatment of a disease, but we do not work on the most emotional aspect of these situations or dealing with the patient's family.” (M64)*

### Provides a Guideline for Acting in Delicate Situations

Both student groups reported that the SS provided valuable *guidance and recommendations* on how to act during end-of-life situations or the final moments of life.

*“Knowing how to act in these difficult, but daily situations and (giving) the security of how to do it right the next time it comes up in practice” (N8).*

*“We had studied these notions in class but knowledge through experience is necessary. At those moments, if you have not been before in a similar scenario, regardless of how much you have studied, it is easy to get blocked and to not know what to do.” (M93)*

### Exercising Introspective Self-knowledge

Students noted that the scenario prompted *introspective reflection on their emotions* in a delicate situation. For some, these were new experiences, and they considered the scenario a valuable exercise for enhancing emotional management.

*“[...](...) The simulated scenario has given me, above all, an opportunity for self-knowledge and reflection.” (M64)*

*“The simulated scenario has fulfilled the objective of transmitting real sensations, making us face feelings, our own and those of others, and to work on their management.” (N74)*

Students reflected on their reactions during the scenario, *recognizing their strengths and weaknesses* to address for improvement.

*“Personally, facing that situation where the patient dies seemed very difficult to me. […] Furthermore, I think that in this way I have discovered a little how I would have acted"spontaneously"and I think that in the future I can use some guidelines that I learned in the workshop. […]” (M77)*

*“Learn how to act in this type of situation and know what capacities I have and which ones I should develop in order to better support the patient and the family” (N72).*

Additionally, students of both professional groups claimed to have *rediscovered PC*.

*“I think it is essential to carry out this simulated scenario because it provides us with a more complete vision of the subject of palliative care […]” (N29)*

*“ […]This workshop has reminded me of that moment [a moment during his/her clinical rotation in a palliative care center where he/she witnessed the death of a patient] and given me a new perspective, as it made me think about the relief that the work of the palliative care doctor brought to the patient and their family, and not just focus on the pain and sadness of that moment.” (M5)*

## Discussion

Our data indicate that medical and nursing students viewed the SS of the LHoL as a positive learning experience. They emphasized its necessity, value, and utility for their future practice, recognizing LHoL as a key aspect of their clinical reality.

Moreover, several of the themes and sub-themes highlighted by the students closely align with the educational objectives established in the Spanish national guidelines for undergraduate training in Medicine and Nursing [13, 14]. They are also consistent with the accreditation standards defined for PC professionals [15, 16] as well as with the recommendations issued by national and international organizations in the field of PC [17, 18]. From both degree perspectives, this training fosters the development of essential clinical competencies such as terminal patient assessment, holistic care, breaking bad news, symptom management, and clinical decision-making in complex scenarios. Additionally, it strengthens key transversal skills including teamwork, empathy, and emotional sensitivity to the suffering and needs of patients and their families.

Both student groups highlighted that the scenario underscored the significance of their role as healthcare professionals and the impact they can have on the patient and family during difficult times. These findings are significant because death is frequently perceived as a failure in medical literature [19], although end-of-life care is part of the ethical codes of medicine and nursing, including patient and family support, limiting diagnostic actions, palliative sedation, etc. [20]

The study results support the notion that simulation is an effective pedagogical strategy for applying theoretical knowledge in a controlled, authentic environment [8, 21–25]. Specifically, it demonstrates that simulation is feasible and beneficial for teaching end-of-life care.

Students recognized that the content of this SS was relevant to genuinely delicate situations, such as the LHoL and death. This finding aligns with existing literature [25, 26], which identifies conversations and support for patients and their families during these final hours as particularly challenging. Teaching care in LHoL is rarely included in medical curricula. Only one study with four medical students involved a hybrid simulation of a patient with terminal lung cancer has been found [24]. Pre- and post-test results indicated improved attitudes towards caring for terminal patients and their families, suggesting that this approach effectively enhances attitudes in the absence of experiential learning. In nursing education, there is slightly more coverage of the topic, with several studies indicating that death scenarios are seen as positive and useful learning experiences [25, 27–29]. These studies emphasize the importance of the support and care nurses should provide to patients and their families, highlighting the greater emphasis placed in nursing education on addressing both patient and family needs. This is consistent with the findings of our study, in which only nursing students referred to the reaffirmation the SS provided in recognizing the value of being present for the patient and offering support to families—an aspect not mentioned by medical students.

Globally, the limited number of studies on teaching care in the LHoL may be attributed to death being a taboo subject both socially and within healthcare contexts [19]. In addition, although cultural norms significantly influence how death is perceived, communicated, and managed, particularly in increasingly multicultural societies, this simulated scenario was intentionally designed to remain culturally neutral. No specific reference was made to religious beliefs, familial traditions, or cultural rituals. The primary aim was to expose students to a general end-of-life situation, emphasizing the recognition of universal signs and symptoms of dying, as well as the communication of this process in a respectful and professional manner. This neutral approach enhances its potential transferability across diverse educational settings. Any necessary adjustments for local implementation would mostly involve communication styles or cultural values that could be discussed during the debriefing phase, rather than the core structure of the scenario itself. In fact, the workshop has been translated into English and included in a European project (E-Learning on Palliative Care for International Students, ELPIS) [30], in which its content and structure were reviewed and endorsed by partners from six different countries. This reinforces the scenario’s relevance and applicability in diverse cultural and educational contexts.

Searches for training on death and dying predominantly yield references to courses on cardiopulmonary resuscitation and emergency care [31]. While some simulations focus on delivering bad news to families following unexpected deaths, this represents only a partial view of the broader realities of death within the healthcare system. The SS in the LHoL carried out in this study offers an alternative perspective on death and highlights the value, usefulness, necessity, and positive impact according to students of such an intervention. Both nursing and medical students emphasized that learning to address the needs and emotions of patients and families, beyond vital signs, is crucial for understanding their responsibilities [29].

Students acknowledged that they will encounter such delicate situations in their careers and found the SS a valuable learning experience. They appreciated the clinical guidance it provided, including recognizing physiological signs of dying and prioritizing comfort, rather than solely focusing on communication skills, as is commonly emphasized in the literature [31–33]. Focusing on knowledge beyond theoretical content, only medical students specifically highlighted the value of the workshop in helping them to gain essential emotional management. This aspect of emotional self-awareness was not explicitly mentioned by nursing students, whose reflections were more focused on responding to the needs of patients and their families. This difference may be attributed to the intense emotional burden experienced by medical students, as many were confronting, for the first time, a highly sensitive situation such as the death of a patient and the task of breaking the news to their family.

Students considered the realism of the SS critical for accurately experiencing and handling the situation. The use of standardized actors as both patient and family member, rather than the typical hybrid scenario with a manikin as a patient and standardized actor as a family member [34–36], was considered beneficial. In another study, nursing students have noted that hybrid scenarios lack realism due to the limited interaction with manikins [34]. In contrast, in our study, the use of standardized actors throughout the SS was noted to have a significant impact, eliciting emotions that encouraged reflection on both theoretical-practical and introspective levels.

This SS could also offer an opportunity for interprofessional team building, a crucial competency in LHoL care [35, 36]. This aspect was highlighted solely by medical students, who found the assistance of nurses during the SS to be highly valuable and emphasized the importance of teamwork. This discrepancy may be attributed to the presence of a nurse in the medicine scenarios, whereas nursing scenarios did not include a presential doctor.

The realism of the SS also facilitated introspection regarding students’ emotional responses and their management. Both medical and nursing students acknowledged that the simulation evoked strong emotions, which is an inherent aspect of caring for patients in the LHoL who are often unconscious and supporting their family as death approaches [37–40].

During the debriefing, medical and nursing students requested strategies for managing emotions, focusing not only on how to deal with those of family members but also primarily on their own. This reflects an initial step towards professional self-awareness, which is increasingly recognized in the literature as essential for developing self-care throughout a clinical career [41, 42]. However, further research is needed to validate and explore the effectiveness of simulation in teaching and reflecting on professional self-care.

The introspection also involved their roles as healthcare students, who felt like authentic professionals during the SS. They deeply contemplated their professional roles as doctors/nurses, gaining a true understanding of their responsibilities, an aspect that has not been found in any other article. For nursing students, it was a confirmation of their role in contributing to patients and families [43, 44], while for doctors, it was more about discovering the human aspect of their profession. They recognized they are humans, not just providers of medical techniques, highlighting the importance of a holistic, empathetic approach to patient care. This underscores the current dehumanization in medicine [19, 45].

This reflective reasoning in Medical and Nursing students suggests that simulations can aid in developing attitudes, not just theoretical and practical skills [29]. It may also contribute to the hidden curriculum, but further studies are needed to confirm this.

*The limitations* of this study include that the research was conducted in one university, which may limit the generalizability of the findings. A 50–67% response rate is good for a one-time contact study. Higher rates might have been achieved with follow-up reminders. Incorporating member checking could help validate the interpretations and enhance the trustworthiness of the findings. While analyzing the quotes from active students and observers separately could have strengthened the results, doing so might have compromised participant anonymity. Medicine students reported shifts in their professional role and perspective. Quantitative assessments of these changes would be valuable. Furthermore, future research should incorporate longitudinal studies assessing the impact of this kind of scenario in the daily clinical practice. It would be interesting to explore the potential of a shared SS of this type to promote interprofessional team building during the LHoL of a patient.

**Fig. 2:**
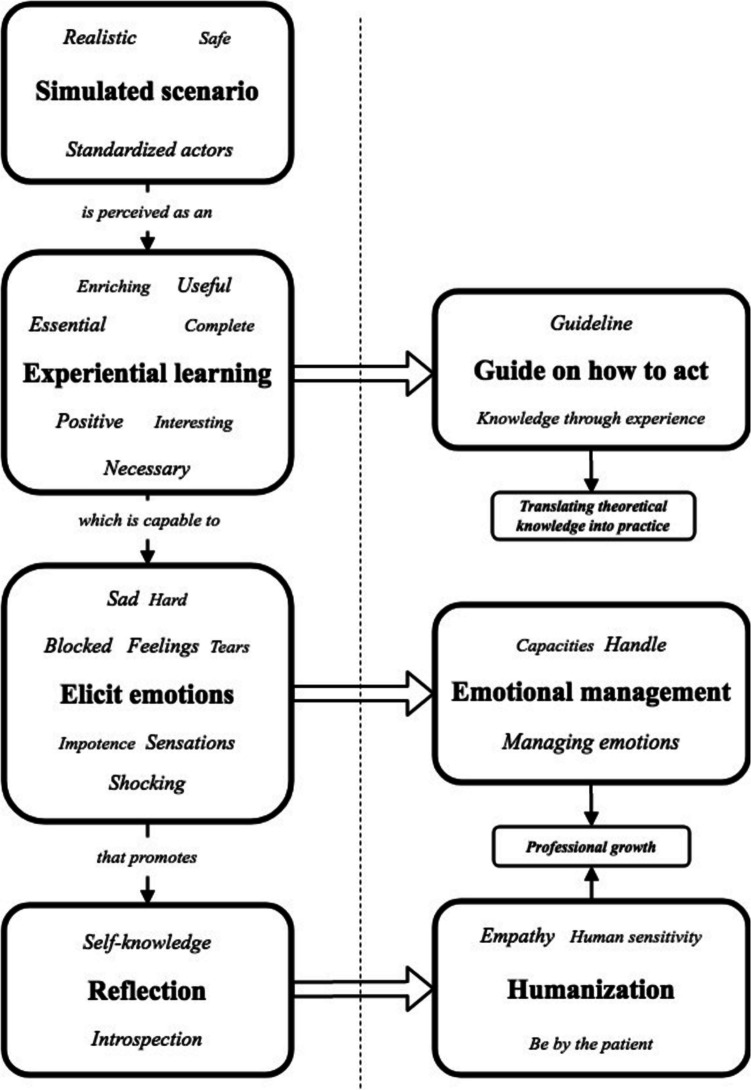
Conceptual flowchart representing the learning process experienced by healthcare students during SS of LHoL. On the left side, the diagram illustrates how the SS fosters experiential learning, which elicits emotions and prompts individual reflection. On the right side, arrows show the learning outcomes—both cognitive and attitudinal—derived from this process, ultimately contributing to professional growth and the application of theoretical knowledge in practice. The words included within each box represent word clouds extracted from students’ qualitative responses; the size of each word reflects its frequency in the comments

*In sum*, Medicine and Nursing students perceived the SS of LHoL with standardized patients as a realistic, positive, and safe learning experience. It allowed them to manage both their own professional self-awareness and patients’ emotions, provided practical tools for navigating sensitive clinical situations, provided useful knowledge for sensitive situations (practical guidelines), and prompted deep introspection about their professional role (Fig. 2).

The insights and competencies reflected by students through this SS are closely aligned not only with the educational objectives of PC, but also with broader learning goals in Medicine and Nursing degrees and accreditation standards. This alignment reinforces the value of the SS as an effective pedagogical tool for fostering essential clinical competencies and transversal skills such as symptom recognition, communication, and holistic care.

Given its educational relevance and clinically oriented design, this SS could be adapted for use in other international contexts, though it remains essential to consider the cultural factors that influence how death is perceived and managed across societies.

A valuable next step would be a longitudinal study using a mixed-methods approach to combine quantitative and qualitative data, aimed at evaluating the long-term impact of this SS on clinical performance and emotional resilience.

## Supplementary Information

Below is the link to the electronic supplementary material.

ESM 1 (19.5 KB DOCX)

ESM 2 (19.8 KB DOCX)

## Data Availability

The data is not going to be able due to ethical restrictions involving a vulnerable population, data access is limited to the principal investigators.gly.
