# Supplementary material for: Learning Last Hours of Life Care Through Patient Simulation Scenario: Experiences of Medical and Nursing Undergraduate Students
Source: Med Sci Educ. 2025 Jul 16;35(5):2451–62. doi: 10.1007/s40670-025-02457-x (PMC12812122; doi:10.1007/s40670-025-02457-x)
Supplement: Supplementary file 1 — (19.5 KB DOCX) [file 40670_2025_2457_MOESM1_ESM.docx]

**WORKSHOP IN THE SIMULATION CENTER: END-OF-LIFE CARE**

Document for the teaching team

*Material to be provided:* document for the participant including an adapted version of the end-of-life clinical care procedure from the University of Navarra Clinic.

*Number of students*: maximum 15 students

*Actors*: 2 actors (a family member and a patient) and a professional support nurse.

**Elements and educational objectives of the scenario:**

The clinical scenario in the medical simulation center for a terminal patient in the final moments of life will encompass the following elements:

1. Introduction: participants will receive a description of the clinical situation, including basic information about the patient, their medical history, and current conditions.
2. Clinical assessment: participants will assess the patient's clinical status, including vital signs monitoring, symptom identification, and treatment response evaluation.
3. Clinical decision-making: participants must make important clinical decisions, such as pain and other symptom management, treatment choices, and communication with family members and other caregivers.
4. Communication with family members: participants must communicate with the patient's family members and provide them with information and support during this difficult moment.
5. Emotional management: participants must handle complex emotional situations and help family members cope with death.
6. Assessment and feedback: after completing the scenario, participants receive feedback on their performance and are encouraged to reflect on their experience.

**DYNAMICS**

1. Introduction: participants will receive a description of the clinical situation, including basic information about the patient, their medical history, and current conditions.
2. SCENE 1 (10-15min)

The first part will be carried out with two volunteers between the students.

These volunteers will read aloud information about the case. They will be informed that they are a home care team consisting of two doctors (the two volunteer students) and a nurse, and that they have received a call from the patient's family member who has provided some information about the case. Before going to the patient's home, they will review the materials they have in their bag with the nurse: pulse oximeter, blood pressure cuff, stethoscope, wound care materials, catheterization supplies, subcutaneous infusion set, and medication (analgesics, diuretics, morphine, antipsychotics).

The rest of the class will have a checklist.

1. Debriefing part 1 (10-15min)

The questions that must guide the debriefing with all the students (volunteers and the rest of the class)

- What happened?
- How did you feel?
- What did you do well?
- What did you find most difficult?
- Ask the patient and the companion how they felt.

After that, with the same volunteers or another two different people, it is going to start the second part.

1. Start SCENE 2 (10-15min)

The rest of the class will have the checklist.

1. Debriefing part 2 (10min)

The questions that must guide the debriefing with all the students (volunteers and the rest of the class)

- What happened?
- How did you feel?
- What did you do well?
- What did you find most difficult?
- Ask the patient and the companion how they felt.

**DEVELOPMENT OF SCENE 1**

**Oncological patient**: characterized by hepatic failure

**Setting:** patient's room. The patient "is catheterized."

**Content of the urine bag:** Coca-Cola (a little), it could be water with betadine

**Room:** bedside table with medication, glass of water, and some furniture (some photos, books..)

**Patient:** painted entirely yellow (visible parts of the body) with pijamas, lying in bed.

**Family member:** wearing street clothes

**Nurse:** real nurse, carries a home care bag: pulse oximeter, blood pressure cuff, stethoscope, wound care materials, catheterization supplies, subcutaneous infusion set, and medication (analgesics, diuretics, morphine, antipsychotics)

**Information for students *(the information that they read before starting the simulation)***

*48-year-old patient, married, 4 children. Diagnosis of metastatic lung adenocarcinoma with massive hepatic progression on the last CT scan a week ago, having exhausted all therapeutic options. Home care was agreed upon with the patient and family.*

*This is the medication he went home with:*

- *Dexamethasone 8mg (pain in the right hypochondrium)*
- *MST extended-release morphine 30mg every 12h (pain in the right hypochondrium)*
- *Morphine rescues, half a 10mg tablet. In case of pain.*

*Yesterday he presented an acute urinary retention and had to be catheterized.*

*A call has just been received from the patient's wife, Carla, saying that this morning she sees him very asleep and he is saying some incoherent things. She is very worried about the situation. She comments that he is so asleep that she could not give him the medication.*

**Comments:**

*To the patient:*

- The patient is not going to die. He is lying in bed, breathing calmly. Eyes closed.
- Frowning gesture, as if in pain, bringing one or both hands to the liver area. Not agitated, but somewhat restless (frequent arm movements, changes in posture, furrowed brow, gestures of pain, sighs or "ayy").
- Upon the call, he opens his eyes, moves confusedly, responds with correct monosyllables to yes-or-no questions (for example, "do you have pain? Do you have difficulty breathing? ...") and to general questions or those not related to symptoms, he responds with short, incoherent sentences (for example “Where is the dog? Why don’t we go shopping?”…). At no time he becomes agitated, but he moves when stimulated. When the stimulus stops, he closes his eyes and keeps moving but less.
- If both the family and the healthcare personnel hold your hand, or caress you, you stop moving, you become comfortable.
- If medication is administered to you, you become comfortable.

*To the family member (wife):*

- The companion is distressed and worried. She is sitting next to the patient, holding his hand with a handkerchief, crying. When the healthcare personnel enter, she stands up, approaches, and explains the situation a bit: "*This morning it was very difficult for him to wake up, he didn't want to have breakfast... I see him very asleep... I didn't dare to give him the medication because I'm afraid he'll choke. Last night he said some incoherent things, like he saw something but I didn't see him stressed or nervous about it.*"
- If the staff tells you to go somewhere, you do it; you are cooperative. If they don't tell you where to go, you return to the bedside and accompany the patient.
- You answer all questions:
  - Drowsiness: since last night you see him more asleep, less talkative... Right after he was catheterized, he was quite restless, he became calmer, more asleep.
  - Hallucinations: you're not sure, but he says many incoherent things.
  - Tremors (they may mention myoclonus, which is the medical term): he hasn't had any. But you see him restless. If they ask about myoclonus, tell them you don't understand what they mean.
  - Food and drink: nothing since last night, but he hasn't asked for any.
  - Defecation: yesterday and very pale, like the last few days.
  - Urine: what they see; if they ask since when it's been like that: since last night.
  - Other questions: nothing new.
- If when they finish talking to the patient they don't speak to you, ask them "What's your opinion, doctor?"
- If they don't mention the restlessness, ask if something can be given to calm him down, because he seems to be suffering.
- If they mention the restlessness but don't say how to administer the medication, ask how they are going to administer it if he has been asleep for a long time and you haven't been able to give him any oral medication,” isn't there another way besides orally? They should tell you that they're going to administer all of it subcutaneously. When they tell you this, if they haven't already mentioned that the pain medication will also be administered subcutaneously, ask them if the pain medication can also be given in that way.
- When they explain that the end is near, sit next to the patient and start crying.
- If they come close to you, return the gesture of affection and ask what we do now.
- If they don't come close, wait a bit, and then ask the same question.
- When they explain everything, express your gratitude and say that you'll call the family if the doctor hasn't suggested it already.

*To the nurse (it is a real nurse):*

- Act as if you were working, but try to let the students time enough to think and act, providing small hints to guide them through the scenario. For example:
  - If they do not propose the subcutaneous route, suggest it to them.
  - If they do not focus on the restlessness, suggest them to administer some medication for it.

**DEVELOPMENT OF SCENE 2**

**Oncological patient** characterized as having hepatic failure

**Setting:** Patient's room. Same as before, plus a bedside table with medication, a glass of water, and some furniture.

**Patient:** Entire body painted yellow (any visible body part) wearing pajamas, lying on the bed.

**Family member:** Wearing casual clothes.

**Information for the students**

*Scene where we left off.*

Routine morning visit. No calls received during the night.

**Comment**s:

*To the patient:*

- Patient, calm, slowly breathing. Doesn't respond to any stimuli.
- Mouth a bit open. When the doctors finish talking to the family member, you start with more pronounced pauses of apnea (stop breathing for a few seconds and then take a slightly deeper breath, moving the chest a little). 3-4 times and then stops breathing.

*To the family member (wife):*

- Carla, calmer than yesterday, but nervous. Emotional lability. When the doctors enter, you approach them with a gesture of gratitude.
- If they don't ask you, express a lot of gratitude for yesterday.
- If they ask you: He has been very calm, only needed rescue medication once to remain calm. He hasn't shown any signs of pain. You have noticed that he breathes more softly and that there are moments when he stops breathing, but then takes a very deep breath and continues breathing. That has happened in the last few hours. You return to the patient's side and hold his hand.
- If the doctors don't ask you anything, ask them *"How do you see him today?"*
- If they ask any other question: Respond that he has been calm but is breathing strangely. If they ask about urine, you haven't seen anything new.
- When the doctor explains you that is the last hours of life and that the patient could pass away soon you gets emotional and goes to the patient's side.
- After the 4 apneas, we assume the patient has passed away.

If the student doesn't realize it, or isn't by your side, ask, *"Has he passed away already?"* Then you get emotional, but very grateful to the team.

- - Maybe is the nurse, if no one says anything, that is going to announced that the patient has passed away.

*To the nurse:*

- While the companion talks to the doctors, the nurse approaches the patient.
- When she returns, she whispers to the doctor (IMMINENT agonal situation)
- After the 4 apneas, we assume the patient has passed away, if no one says anything, says that the patient has passed away.
