# Supplementary material for: Learning Last Hours of Life Care Through Patient Simulation Scenario: Experiences of Medical and Nursing Undergraduate Students
Source: Med Sci Educ. 2025 Jul 16;35(5):2451–62. doi: 10.1007/s40670-025-02457-x (PMC12812122; doi:10.1007/s40670-025-02457-x)
Supplement: Supplementary file 2 — (19.8 KB DOCX) [file 40670_2025_2457_MOESM2_ESM.docx]

**Clinical Scenario: Final Hours (Nursing)**

**Part I:**

- 1. **Synopsis (INFORMATION FOR NURSES)**

Pablo, a 35-year-old patient, married to Carla, with two children. Diagnosed with metastatic lung adenocarcinoma with massive hepatic progression on the last CT scan one week ago, after completing all available therapeutic options. Home care was agreed upon with the patient and the family.

The doctor visited yesterday and informed the family that the patient is in his final weeks.

This is the medication he is taking at home:

- Dexamethasone 8 mg (pain in the right hypochondrium).
- MST prolonged-release morphine 30 mg every 12h (pain in the right hypochondrium).
- Morphine rescue doses, half a 10 mg tablet, in case of pain.
- Laxative due to morphine.

Yesterday morning, he presented with acute urinary retention and had to be catheterized.

The nurses receive a call from the patient’s wife, Carla, saying that this morning he seems very sleepy and he has said some incoherent things. She is very worried about the situation. She says he is so drowsy that she has not been able to give him the pain medication.

**INSTRUCTIONS FOR NURSES**

The nurses (students) may call the doctor to consult about the patient’s current condition and the possibility of changing the medical order to adapt it to the patient’s condition and needs.

The route of administration of medications can be changed, simulating the administration.

**1.2 Actor Preparation and Simulated Home Environment**

- 35-year-old patient with hepatic failure secondary to oncologic process.
  - The patient has a urinary catheter with minimal, cola-colored output.
  - The patient presents with jaundice, visible on all exposed areas of the body, and is lying in bed wearing pajamas.
- Setting: the patient's room at home.
  - Room: nightstand with medication, glass of water, chair for the companion, and some furniture (if available).
- Companion: wearing street clothes.

**1.3 SCENARIO**

Patient (Pablo): He will not die during the scenario. He is lying in bed with calm/normal breathing, eyes closed. He maintains a pained expression (frowning, occasionally clenches his mouth…), places one or both hands on his liver area. He is agitated, somewhat restless (frequent arm movements, posture changes, frowning, expressions of pain, sighs or “ayy”).

When addressed, he opens his eyes, moves confusedly, and answers yes/no questions correctly with monosyllables (e.g., Do you have pain? Are you having trouble breathing?). For general or non-symptom-related questions, he responds with short incoherent phrases. When the stimulus stops, he closes his eyes.

The companion (his wife, Carla) looks distressed and worried. She is sitting next to the patient, crying with a tissue. She holds his hand most of the time. When healthcare personnel enter, she stands up, approaches, and describes the situation:

*“This morning he had a hard time waking up, didn’t want breakfast… he seems very sleepy… I didn’t dare to give him the medication because I’m afraid he’ll choke and I think he’s in some pain.”*

If staff tell her where to go, she complies. If they don’t, she returns to the bedside and stays with the patient.

She responds to all questions or, if the students do not inquire, gradually provides information about the patient's condition.

- Drowsiness: Since last night, more sleepy, less talkative...
- Yesterday morning he was a bit restless before being catheterized but then calmed down, “like more sleepy.”
- Food and drink: none since last night, but he hasn’t asked for any.
- Stools: yesterday, very pale, as in recent days.
- Urine: what’s visible; if asked since when: since the night.
- Other questions: nothing new.

If students do not speak to the companion after assessing the patient, she should ask: “What do you think? How do you see him?”

If nothing is said about the medication, she asks: “What should we do about the pain meds?” If they don’t suggest changing the route, she may ask: “Is there another way to administer it? Could you speak to the doctor?” Nurses (students) should call the doctor to consult on the current condition and possible adjustments to the medical orders.

When they explain to the wife that the prognosis seems poor based on symptoms, she sits beside the patient and starts crying.

- If they approach her, she responds to their gesture and asks: “So what now? What’s the new plan? What should I expect from now on?”
- If they don’t approach, wait a bit, then ask the same.

If they don’t answer, suggest meeting with the doctor.

If they do not provide further guidance on how to care for the relative or how to be involved in their care, ask for it: “What more can I do for him? Can I help care for him somehow? Should I call someone?”

When everything is explained (need to consult the multidisciplinary team, assessment by a doctor, the planned course according to the patient's current condition, etc.), she thanks them and says she will call the family if not already suggested.

If the staff do not leave, she tells them they can go if they wish and that she will call if things worsen.

**END OF SCENARIO**

**1.4 Debriefing Part I**

**General aspects**

Ask the volunteers:

- How did you feel?
- What did you do well?
- What could have been done differently?
- What was most difficult?

Ask the observers for positive and improvable aspects.

**Part II:**

**2.1 Synopsis (this information is provided to the students prior to the scenario)**

We continue with Pablo (the same patient from the previous scene). The nurses visit the home the following morning as part of a routine visit. The family has already been informed by the physician that Pablo's prognosis is that he is expected to die within hours or days. Carla feels overwhelmed by the situation and refuses to leave Pablo’s side, as she doesn’t want him to go without her being present.

**2.2 Actor Preparation and Simulated Home Environment**

- 35-year-old patient with hepatic failure secondary to oncologic process (same patient).
  - Still catheterized: the patient has a urinary catheter with minimal, cola-colored output.
  - The patient presents with jaundice, visible on all exposed areas of the body, and is lying in bed wearing pajamas (same as before).
  - A subcutaneous butterfly needle has been added to an exposed area of the body if it wasn’t already placed in the previous scenario.
  - Setting: the patient’s room at home.
- Room: nightstand with medications, glass of water, chair for the companion, and some furniture (if available).
- Companion: wearing street clothes.

**2.3 Actor Notes and Scenario Development**

Carla is uneasy and overwhelmed. Nervously, she comments about the patient:

*“After you gave him the medication yesterday, he became very calm. He was even lucid, and I gave him a spoonful of gelatin. At night, he became restless again. I gave him several rescue doses for the pain, but they didn’t work... So I followed your advice and gave him the medication (haloperidol) to calm him down… I didn’t know what to do… I didn’t want to bother you… but I was feeling so anxious… I didn’t know if he was suffering… I just wanted him to be calm in these last moments… and now he’s so drowsy, he doesn’t respond to my stimuli… Was it because of what I gave him? Oh my God, I only wanted to help him not suffer… Did I do something wrong?”*

She stays next to the patient the entire time, and with every movement he makes, she tries to hold him gently. If the students ask about what has changed, she explains that since administering the medication for agitation last night, he has been very drowsy and she has the impression that he occasionally stops breathing. She may mention that some family members have come to say goodbye.

Pablo sleeps calmly. Apneas appear intermittently. He does not respond to any type of stimulus.

Allow the nurses (students) to assess the patient and realize that death is imminent, with increasingly prolonged apneas, etc. Give them time to communicate this reality in some way to the companion.

Carla begins to move the patient and sees he does not respond. She says he isn’t breathing and begins crying inconsolably, making it apparent that the patient has died. At this point, the nurses (students) should approach the bedside to confirm the death, offer condolences, etc. After a suitable amount of time, the students should begin to provide post-mortem explanations (body preparation, funeral home, etc.). If they do not, the companion should ask: “What should we do now?”

After the appropriate explanations, Carla will request a moment to say goodbye for the last time and will indicate that they may enter to prepare Pablo afterwards.

**END OF SCENARIO**

**2.4 Debriefing Part II**

**General aspects**

Ask the volunteers:

- How did you feel?
- What did you do well?
- What could have been done differently?
- What was most difficult?

Ask observers: positive aspects and areas for improvement.

Ask the patient and companion how they felt.
